# Comparative Anatomy of Phagocytic and Immunological Synapses

**DOI:** 10.3389/fimmu.2016.00018

**Published:** 2016-01-28

**Authors:** Florence Niedergang, Vincenzo Di Bartolo, Andrés Alcover

**Affiliations:** ^1^U1016, Institut Cochin, INSERM, Paris, France; ^2^UMR 8104, CNRS, Paris, France; ^3^Université Paris Descartes, Sorbonne Paris Cité, Paris, France; ^4^Lymphocyte Cell Biology Unit, Department of Immunology, Institut Pasteur, Paris, France; ^5^U1221, INSERM, Paris, France

**Keywords:** phagocytosis, immunological synapse, immune receptor, signal transduction, actin, microtubules, exocytosis, endocytosis

## Abstract

The generation of phagocytic cups and immunological synapses are crucial events of the innate and adaptive immune responses, respectively. They are triggered by distinct immune receptors and performed by different cell types. However, growing experimental evidence shows that a very close series of molecular and cellular events control these two processes. Thus, the tight and dynamic interplay between receptor signaling, actin and microtubule cytoskeleton, and targeted vesicle traffic are all critical features to build functional phagosomes and immunological synapses. Interestingly, both phagocytic cups and immunological synapses display particular spatial and temporal patterns of receptors and signaling molecules, leading to the notion of “phagocytic synapse.” Here, we discuss both types of structures, their organization, and the mechanisms by which they are generated and regulated.

## Introduction

**Figure 1:**
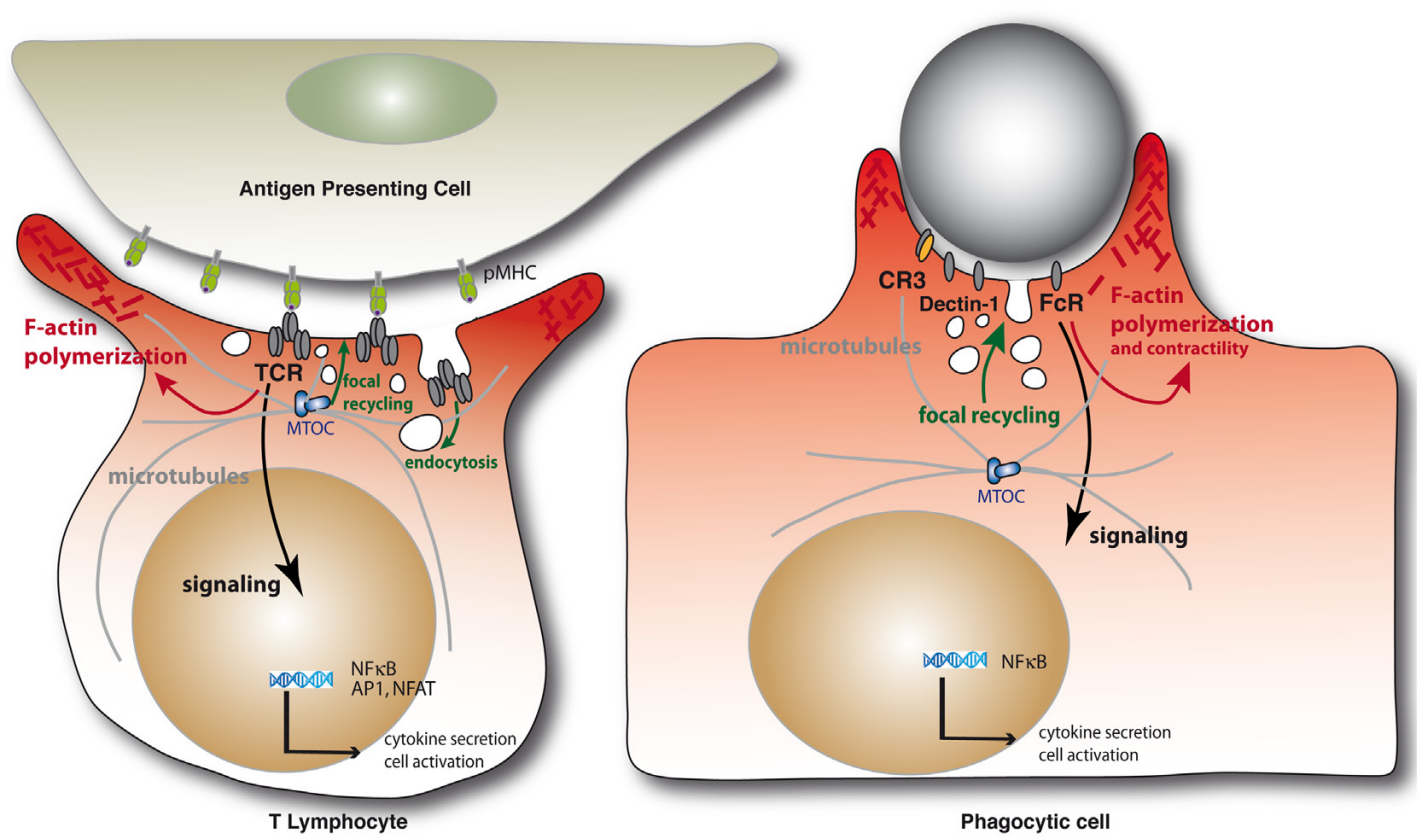
**Schematic representation of the immunological synapse and the phagocytic cup formation**. Immunological synapse formation is initiated by the engagement of TCRs on the surface of the T lymphocytes by peptide antigen–MHC complexes on the APC (left). Similarly, engagement of phagocytic receptors by multiple ligand binding on a target particle drives the formation of phagocytic synapses (right). In both settings, receptor engagement leads to F-actin polymerization and membrane deformation at contact sites. Polarization of the MTOC and microtubule network toward at the IS are important for the delivery of vesicles containing cytokines or lytic enzymes in helper or cytotoxic T cells, respectively, but also to deliver TCR-signaling components during immunological synapse formation. Microtubules also contribute to F-actin remodeling in complement-mediated phagocytosis. Internalization of cell surface TCRs by endocytosis and their focal recycling participate in the regulation of T cell activation. Finally, in either system, triggering of multiple signaling pathways downstream of the surface receptors leads to *de novo* transcriptional programs controlling cell survival, activation, and cytokine production.

Immunological synapses are organized cell–cell contacts shaped at the interface between T cells and antigen-presenting cells (APCs) (Figure 1). They are triggered by the binding of T cell antigen receptors (TCR) to their ligands, peptide antigens associated with major histocompatibility complex molecules (pMHC) expressed on the surface of APCs. TCR engagement induces the polarization of the T cell toward the APC and a coordinated reorganization of various T cell components, including receptors, signaling and adhesion molecules, the actin and microtubule cytoskeleton, and intracellular vesicle traffic. Thus, the TCR and its proximal signaling molecules (e.g., protein kinases and phosphatases, signaling adapters, and effectors molecules) form dynamic signaling complexes at the immunological synapse that drive T cell activation. Moreover, TCR signaling triggers the fine reorganization of the actin and microtubule cytoskeleton that ensures synapse architecture and signaling complex dynamics, critical for TCR signal regulation. Finally, various intracellular compartments polarize toward the immunological synapse, including the Golgi apparatus, early and late endosomes, and mitochondria. Importantly, the TCR signaling machinery, actin and microtubule cytoskeleton, and intracellular vesicle traffic interplay at the synapse to sustain and regulate T cell activation (1).

Phagocytic cup formation mirrors a large number of events occurring during immunological synapse formation, before leading to a productive engulfment of the target (Figure 1). First, clustering of phagocytic receptors induced by particle-associated ligands triggers signal transduction pathways similar to those engaged by the TCR. In particular, a similar spatial and temporal segregation of tyrosine kinases and phosphatases was observed at both immunological synapses and phagocytic cups, leading to the notion of “phagocytic synapse” (2). Second, phagocytic receptor signaling triggers a profound reorganization of the actin cytoskeleton that is similar to the one induced by the TCR, generating large membrane extensions rich in filamentous (F)-actin. Third, microtubule dynamics are also important for some receptor-mediated phagocytosis. Fourth, intracellular traffic involving several vesicular compartments reorients toward the phagocytic cup. Fifth, internalization of the triggered receptors together with their ligands occurs and may lead to their degradation or recycling back to the plasma membrane. Interestingly, the TCRs may be phagocytosed from the immunological synapse internalizing with them their pMHC ligands together with portions of the APC membrane (3). Finally, a series of downstream signaling events lead to cytokine gene activation in both cases.

We review here the molecular and cellular events taking place in both phagocytic and immunological synapses, highlighting their mechanisms of regulation.

## Receptor Signal Transduction in T Cells and Phagocytes

T cell receptor engagement induces a series of molecular reorganization events that stabilize T cell–APC interactions and optimize signal transduction. Several other receptors are recruited to the immunological synapse and contribute to the activation process. These include the co-receptors CD4 and CD8, co-stimulatory receptors such as CD28, or adhesion proteins such as the integrins α_L_β_2_ (LFA1) or α_4_β_1_ (VLA4) [reviewed in Ref. (4)].

One of the earliest events elicited by antigen recognition is the sequential activation of protein tyrosine kinases belonging to the Src and Syk families. The Src-family kinases Lck and/or Fyn, phosphorylate several TCR complex subunits, namely CD3 (ɛ, γ, and δ) and ζ (5). These subunits are all endowed with one or more consensus sequences called immunoreceptor tyrosine-based activation motif (ITAM), each containing two phosphorylatable tyrosine residues. Doubly phosphorylated ITAMs then recruit Syk-family kinases, either ZAP-70 or Syk (6), whose tandem SH2 domains provide specific, high-affinity binding to ITAM phosphotyrosines. Src kinases may be further required to phosphorylate and activate Syk kinases, in particular ZAP-70. The interplay between these two families of tyrosine kinases is crucial for transmitting downstream signals. Thus, Syk family kinases phosphorylate adaptor proteins, such as LAT and SLP-76 that in turn gather signaling effectors within multiprotein complexes, or signalosomes (6). Moreover, both Src- and Syk-family kinases activate several enzymes recruited in these signalosomes that are responsible for the generation of intracellular second messengers, such as Ca^2+^ or phosphoinositides. Collectively, these early steps, induced within seconds after TCR engagement, initiate a cascade of downstream events leading to cytoskeletal rearrangement and cellular polarization. Concomitantly, various serine–threonine kinases, including MAP kinases, are activated, regulating the activation of several transcription factors that will drive in turn T cell growth and differentiation and the production of effector cytokines (7).

Detection and engulfment of bacteria or fungi by phagocytic cells are triggered by a similar sequence of early events. However, multiple unrelated ligands trigger phagocytosis by engaging distinct receptors. Indeed, phagocytic receptors can recognize their target by binding either to specific molecules expressed on the target’s surface or to opsonizing antibodies or complement subunits previously bound to the target. For instance, phagocytosis of IgG-coated pathogens is triggered upon antibody recognition by Fcγ receptor (FcγR), whereas integrins, such as α_M_β2 (also known as Mac-1 or CR3), can recognize complement-coated particles. Finally, phagocytosis of fungi expressing β-glucans on their cell wall is triggered by Dectin-1 receptor (8).

Phagocytic Fc receptors (FcγRII and FcγRIII) belong to the immunoreceptor family and are structurally related to antigen receptors. Importantly, they transmit activating signals using ITAM motifs that are either built in the receptor intracellular tail or in the associated common γ-chain (9). Hence, early signaling events involve Src- and Syk-family kinases, similarly to what explained above for the TCR. In macrophages, the Src kinases Lyn, Hck, and Fgr are involved in FcR-induced phagocytosis. However, phagocytosis was significantly reduced but not abolished in cells of triple knockout mice, suggesting the existence of further redundancy or alternative triggering mechanisms (10). In contrast, Syk knockout resulted in a complete block of phagocytosis, indicating the indispensable role of this kinase (11). Since Syk, but not ZAP-70, has been shown to phosphorylate ITAM motifs (12), it can be envisaged that Syk can trigger some phagocytic activity in the absence of Src kinases.

The β-glucan receptor Dectin-1, a member of the C-type lectin receptor (CLR) family, also induces sequential activation of Src and Syk kinases. Dectin-1 displays in its cytoplasmic domain ITAM-like sequences named hem-ITAM, each containing a single tyrosine-based motif. Once phosphorylated by Src kinases, they are able to bind Syk and trigger downstream activation (13). Since Dectin-1 is a dimer, it has been proposed that Syk binds in trans to two phosphorylated hem-ITAMs on adjacent subunits in order to be recruited to the activated receptor (13). However, this model has not been validated experimentally. Furthermore, a potential alternative mechanism for Syk recruitment has been revealed recently, implying a scaffolding role of the protein tyrosine phosphatase SHP-2 in bridging Syk to Dectin-1 and other CLRs (14).

The molecular mechanisms underlying integrin-dependent phagocytosis, such as that elicited by complement-coated particles binding to CR3, are more complex than those described for FcRs and Dectin-1. Importantly, integrin binding to their ligand requires prior activation *via* a conformational change induced by “inside-out signaling.” This priming phase is induced by inflammatory or pathogen-specific signals, such as those triggered by G-protein-coupled (GPCRs) or toll-like receptors (TLRs). These proteins initiate different signaling cascades converging on a common effector, the GTPase Rap1 (15). Active Rap1 induces the recruitment of RapL, RIAM, and talin to integrin cytoplasmic tails, thus promoting the switch of integrins to their extended conformation that can bind ligands with high affinity (16). Then, ligand-bound integrins transmit “outside-in” signals that drive actin polymerization and downstream activation. These steps involve several effectors including the protein kinases FAK (or Pyk2) and ILK, non-muscle myosin II, and Rho GTPases (17). Nonetheless, the fact that Syk inhibition impairs CR3-mediated phagocytosis demonstrates the existence of some crosstalk between integrin activation and ITAM-bearing receptors or adaptors (18). Interestingly, FcRs have confined mobility in the plasma membrane, in fenestrated cortical actin structures that depend on the activity of Src- and Syk-family kinases (19). Integrins or pattern recognition receptors, such as the scavenger receptor CD36, are potentially initiating Syk activation, leading to FcR increased mobility and engagement (8). However, further work is needed to define the molecular basis of integrin interplay with ITAM-dependent signaling.

## Spatiotemporal Organization of Immune and Phagocytic Receptors and Their Signaling Machineries

How early signals are elicited by antigen or phagocytic receptors engagement is still a matter of debate. One model proposed for TCR activation postulates that initial triggering is achieved when key inhibitory proteins, such as the tyrosine phosphatase CD45, are segregated away from the engaged TCR and the proximal tyrosine kinase Lck. This segregation is mainly driven by the size of membrane protein ectodomains. Indeed, the length of the TCR–pMHC pairs is relatively small (7 nm) compared to that of CD45 ectodomain (28–50 nm); hence, TCR engagement by pMHC induces the formation of areas of close juxtaposition of T cell and APC membranes from which phosphatases are excluded (20, 21). As a consequence, local activity of tyrosine kinases would be favored, leading to an increase in net phosphorylation of TCR downstream effectors and T cell activation. Interestingly, a similar mechanism was observed during Dectin-1-dependent phagocytosis, leading to the “phagocytic synapse” model. Indeed, Dectin-1 engagement by β-glucan-bearing particles results in local exclusion of phosphotyrosine phosphatases CD45 and CD148 from receptor-enriched areas containing phosphotyrosine, thus triggering downstream signaling (e.g., Syk phosphorylation) and phagocytic cup formation (2). Importantly, several results suggest that this mechanism also concerns FcRs (22, 23); hence, it may be relevant for all phagocytic receptors.

Concomitantly to initial kinase and phosphatase segregation, T cell receptor subunits, the tyrosine kinases Lck and ZAP70, and the adapters LAT and SLP76 associate into dynamic signaling complexes that nucleate at the periphery of immunological synapses and then migrate toward its center, where they concentrate or vanish (24–26). Interestingly, centripetal dynamics of signaling complexes at the immunological synapse and their concentration in the center is a regulatory mechanism that depends on actin and microtubule cytoskeleton and is meant to downregulate proximal TCR signaling (27–29). Various mechanisms have been proposed for TCR signal downregulation at the synapse. These include relocalization to membrane regions containing the tyrosine phosphatase CD45 (28), internalization and degradation of TCR and signaling complexes (30–32), post-translational modification of signaling adapters leading to signalosome disassembly (33), or the extracellular release of vesicles containing TCR (34). Of note, in FcR-mediated phagocytosis, receptors are engaged sequentially in a receptor-guided, zipper-like advance of the membrane over the particle surface, and there is no evidence for a movement of the receptors toward the base of the phagocytic cup. Receptors are downregulated from the surface with the engulfment of the particle. Thus, the late events in the mature immunological synapse differ from those observed in phagosome completion and closure.

## Actin and Microtubule Cytoskeleton Interplay

Signaling downstream of the TCR and phagocytic receptors leads to intense and transient actin polymerization that relies on the activation of Rho family GTPases (35). In T cells and phagocytes, Rho GTPase activation occurs to a large extent *via* tyrosine phosphorylation and activation of the Rac1 and Cdc42 guanine exchange factor (GEF) Vav (36, 37). In addition, Rac1 can be activated by other GEFs, including DOCK2, DOCK8, Tiam1, and Trio. DOCK2 is involved in Rac1 activation downstream of the TCR and in lymphocyte migration in response to chemokines. DOCK2 and DOCK8 physiological relevance has been underscored by the discovery of human-inherited immunodeficiencies caused by *DOCK2* or *DOCK8* gene mutations. B and T cells from these patients display impaired actin polymerization and migration in response to chemokines, as well as impaired lytic granule release by NK cells (38, 39). DOCK family proteins are also involved in phagocytosis as regulators of Rac1 (40).

In phagocytes, the pioneering description of the involvement of Rho family proteins initially led to the classification of type I phagocytosis implicating Rac1 and Cdc42 downstream of FcR and type II phagocytosis relying on RhoA downstream of CR3 (41). More recently, RhoG has been shown to act as regulator for both FcR and CR3-mediated phagocytosis (42). As RhoG is also critical for phagocytosis of apoptotic bodies (43), and for the nibbling of MHC-associated portions of APC membrane by T cells (3), it could well act as a still ill-defined “master regulator” in immunological synapse and phagosome formation. Dynamic studies by fluorescence resonance energy transfer (FRET) revealed different patterns of activation for Rac and Cdc42 downstream of FcR. Active GTP-Cdc42 is present at the tip of the advancing pseudopod where it colocalizes with polymerizing actin, while Rac1 activation is biphasic. GTP-Rac1 is induced at a low level early after particle binding and peaked at the time of pseudopod fusion (44). Cdc42 activation and phosphatidylinositol-4,5-bisphosphate PI(4,5)P_2_ accumulation in the nascent phagocytic cup activate effectors among which the actin nucleation promoting factor (NPF) N-WASP that acts on the Arp2/3 actin nucleation complex. Rac1 is then essential for F-actin polymerization to complete extension and closure, through activation of another NPF, the WAVE complex. In CR3-mediated phagocytosis, RhoA is critical for the signaling to actin polymerization as it activates the Rho-Kinase (ROCK), the formin mDia1, and myosin II that are implicated in polymerization and contraction of F-actin around the particles (41, 45–47). The microtubules are important for this pathway, and CLIP1 (CLIP-170), a microtubule plus-end protein, is especially required for efficient recruitment of mDia1 downstream of CR3 and therefore for efficient phagocytosis (48, 49), showing crosstalk between microtubules and actin.

Immunological synapse formation and function require the coordinated activation of RhoA after initial LFA-1 clustering and Rac1 and Cdc42 activation downstream of the TCR (35). Active Cdc42 and its effector WASP are independently recruited to the synapse. WASP seems not to be necessary for broad actin polymerization at the synapse, but rather for the generation of dynamically polymerizing actin foci that facilitate PLCγ activation and calcium flux (50). Consistently, WASP is necessary for efficient IL2 production (51, 52). In contrast, WAVE2, Arp2/3, and the cortactin homolog HS1 are required for T cells to regulate actin polymerization at the synapse (53–55). In turn, actin dynamics is necessary for triggering and sustaining T cell activation (56). This occurs in various concomitant ways, including the regulation of T cell–APC conjugate formation *via* integrin clustering (57), the interplay between actin cytoskeleton regulators and the calcium second messenger (58), or the regulation of immunological synapse architecture and its interplay with the TCR signaling machinery (59). Finally, the formation of signaling microclusters around the synapse periphery and their convergence toward the center depends on actin dynamics and F-actin inward flows (24, 60).

Cortical actin-associated proteins, such as ezrin and moesin, play important roles in building an activation competent immunological synapse. These proteins connect the cortical cytoskeleton with membrane components. Thus, moesin supports CD43 exclusion from the center of the synapse, a mechanism proposed to remove the CD43-dependent steric inhibition and to facilitate synapse formation (61–63). Moreover, ezrin and moesin contribute to the architecture of the immunological synapse, cell cortex rigidity, and T cell activation as well as differentially regulate early and late activation events (64–67).

Microtubules are finely reorganized at the immunological synapse bringing the microtubule-organizing center (MTOC) close to T cell–APC contact (67–69). Microtubule polarization depends on TCR-induced signaling (70, 71) and the microtubule-driven molecular motor dynein (72). Interestingly, ezrin plays a critical role in driving the MTOC close to the synapse, in controlling microtubule network organization, and in signaling microcluster dynamics at the synapse. Ezrin does so *via* its association with the polarity regulator Dlg1 (67). Moreover, the actin-nucleating proteins Diaphanous 1 (mDia1) and formin-like 1 (FMNL1) are also necessary to polarize MTOC to the synapse (53). The involvement of ezrin and formins in MTOC polarization highlights that actin and microtubule network organization at the synapse are tightly connected. Microtubule stability modulated by the HDAC6 deacetylase is also regulated during immunological synapse formation and necessary for synapse formation and T cell activation (73). Actin and microtubule interplay is also critical for T cell effector function, such as polarized secretion of helper cytokines, since it is necessary for Golgi complex polarization toward the APC (74).

As mentioned above, microtubule–actin interplay is also necessary for efficient phagocytosis (48). Of note, the MTOC has also been reported to be relocated at the site of phagosome formation (75), but given that multiple targets are often phagocytosed at the same time, how this applies to uptake in physiological situations is uncertain. Similarly, when a cytotoxic T cell is engaged in multiple contacts, the antigen-specific delivery of lytic granules occurs independently of centrosome positioning (76).

Microtubule dynamics and organization ensure the delivery of TCRs and signaling molecules to the synapse *via* recycling endosomes (77–79). Moreover, microtubules, together with actin flows, drive signaling microcluster centripetal movement at the synapse (67, 80). Therefore, microtubules drive the arrival and removal of TCRs and signaling molecules in a way to sustain and regulate TCR signaling at the synapse.

## Actin Dynamics and Clearance

Actin polymerization is crucial to achieve efficient pseudopod extension and phagosome formation, but actin turnover and depolymerization is as important. This turnover, which occurs at the base of the phagocytic cup (81), is directly dependent on the hydrolysis of PI(4,5)P_2_ (82), which is mediated by several effectors including phosphatases that hydrolyze PI(4,5)P_2_, such as phospholipase C, PI3 kinase, and 5′ phosphatases, such as Inpp5b or oculocerebrorenal syndrome of Lowe (OCRL) (81, 83–85). In addition, the severing protein cofilin is recruited to the site of phagocytosis and its activity is regulated by LIM kinase (86). Interestingly, the presence of OCRL at sites of phagocytosis was shown to depend on vesicular recruitment of AP1 and EpsinR adaptors, which is under the unexpected control of the NF-kB signaling protein Bcl10 (81), showing how interconnected the signaling and trafficking events are. Inactivation of Rho GTPases is also achieved by several Rho GAP proteins, such as ARHGAP12, ARHGAP25, and SH3BP1, that are recruited under the dependence of PI3K and synergistically inactivate Rac and Cdc42 (87). Actin clearance from the base of the phagocytic cup, which is required for large but not small particle internalization (87), is then necessary for vesicles to make their way to the plasma membrane (81).

Actin clearance is also observed in immunological synapses, and it is thought to be important to facilitate vesicle fusion at the synapse, particularly in cytotoxic T cells, which destroy target cells by the polarized secretion of lytic granules (88). F-actin relocalization at the immunological synapse depends on PI(3,4,5)P_3_ (89) and modulates cytotoxicity. Actin and PI(4,5)P_2_ are cleared from the site of secretion, indicating a tight interplay between actin cytoskeleton reorganization and phospholipid second messenger at the synapse (68, 90).

Therefore, the reorganization of the actin and microtubule cytoskeleton is triggered by TCR and phagocytic receptors and is the key to maintain the structure and function of phagocytic cups and immunological synapses.

## Vesicle Traffic During Phagocytic Cup and Immunological Synapse Formation

Phagocytic cup formation generates membrane protrusions capable of engulfing particles of different sizes. Instead of a decrease in membrane surfaces after internalization of the phagosomes, an increase in cell surface was reported during phagosome formation using capacitance measurements (91). This is in agreement with the concept of membrane remodeling and “focal delivery” of intracellular compartments at the site of phagosome formation (92, 93). The requirement for focal vesicle fusion in optimal phagocytosis of large targets came from studies interfering with the fusion machineries composed of soluble *N*-ethylmaleimide-sensitive factor attachment protein receptors (SNAREs). These are membrane fusion regulatory proteins that form a tri-party complex composed of one vesicle (v)-SNARE and two target membrane (t)-SNAREs. SNARE complex formation helps bringing together the two membranes to facilitate their fusion. SNAREs act with various regulatory proteins, such as Rab GTPases, Munc proteins, and the calcium sensors synaptotagmins to bring together, dock, tether, and fuse vesicles with target membranes, either the plasma membrane or other vesicles (94). Several intracellular vesicular compartments have been implicated in focal recruitment and fusion concomitant with phagosome formation (95–97). These include recycling endosomes bearing the v-SNARE VAMP3 on their surface (98–100) and late endocytic compartments displaying the v-SNARE VAMP7 or lysosomes (101, 102). The endocytic compartments also harbor the adaptor proteins AP1 and EpsinR, both implicated in efficient phagosome formation, while the AP2 complexes and the clathrin-related endocytic machinery are not involved (81, 100). Interestingly, VAMP3^+^/AP1^+^ endosomes also partially colocalize with TNFα, a cytokine that is delivered at the site of forming phagosomes (103).

Similarly, different endosomal compartments and vesicle traffic regulators are involved in immunological synapse formation. These compartments differentially transport TCRs, the tyrosine kinase Lck, and the adapter LAT to the synapse by recycling these proteins back and forth between their plasma membrane location and endosomes. These endosomal compartments display different traffic regulators, such as Rab GTPases (i.e., Rab4, Rab8, Rab11, Rab27, and Rab35), transport proteins (i.e., MAL, intraflagellar transport proteins), or vesicle fusion regulators (i.e., VAMP3, VAMP7, Synaptotagmin-7, and Munc13) (77, 78, 104–108). The immunological synapse clusters the t-SNAREs SNAP23 and syntaxin 4 preparing the zone for active vesicle fusion activity. It is still a matter of debate whether vesicles transporting the signaling adapter LAT only dock and stay as subsynaptic vesicles (106, 109, 110) or fuse with the plasma membrane driving LAT clustering at the synapse (77, 78, 111, 112). The regulated exocytosis of vesicular compartments in T cells might also be important during the early stages of synapse formation when a large lamellipodium-like membrane structure is formed over the APC. Finally, vesicle traffic is important for T cell effector functions, such as polarized secretion of cytokines or cytotoxic granules in helper and cytotoxic cells, respectively (88, 113).

During phagosome formation, the recruitment of compartments and their fusion are regulated by small GTPases of the Rab and ARF families. Rab11, localized on the recycling compartments, is implicated in efficient phagocytosis (114–116). ARF6 was shown to be activated during phagosome formation and to control the delivery of VAMP3^+^ recycling endosomes (99, 117, 118). Rab35 regulates actin-dependent phagosome formation by recruiting ACAP2, an ARF6 GTPase-activating protein (119), or by regulating the localization of Rac1 and Cdc42 (120). In addition, Rab11 and ARF6 activities might be coordinated *via* common effectors, such as the Rab11-FIP3/4/RIP/RCP (Rab-coupling proteins), also named arfophilins, which were implicated in phagosome formation and maturation (115). Rab31 (Rab22b) recruits the adaptor APPL2 that participates in PI3K/Akt signaling and phagosome completion (121). As Rab35 recruits the OCRL phosphatase during cytokinesis (122), it could also be implicated together with Rab5 (85) in OCRL recruitment during phagocytosis, although this has not been demonstrated. There are therefore multiple levels of regulation that implicate tight coordination between the signaling platforms and their subcellular localization, and further investigations are required to dissect them both in the context of the immunological synapse and the phagocytic cup.

## Conclusion

Although we have largely progressed in our understanding of the mechanisms underlying the membrane and cytoskeletal reorganization that support phagosome and immunological synapse formation, there are still a number of issues that need further in-depth investigation. These issues may be different in the phagocytosis and the immunological synapse fields, but a comparison of the two systems may help solve these different questions faster. These include how some phagocytic receptors get engaged and the type of signals they generate? What is the phospholipid chemistry of each of the systems and its influence on cytoskeleton organization? What is the precise time and space organization of signaling complexes and vesicular compartments? Interestingly, we have recently described several examples of “*ménage à trois*” between receptor signals, vesicle traffic, and cytoskeletal structures in both processes; for instance, the involvement of the proinflammatory signaling pathway NFκB in the control of vesicle trafficking and actin clearance in nascent phagosomes *via* the signaling protein Bcl10 (81), or the orchestrated action of the TCR signaling machinery, the actin and microtubule cytoskeleton, and intracellular vesicle traffic in ensuring immunological synapse architecture and function in T cell activation and effector functions, such as polarized secretion of cytokines or cytotoxic granules (1). Collectively, the vast majority of data presented here emphasize the similarities between immunological and phagocytic synapses formation and suggest a possible evolutionary link between these two structures, whereby the phagocytic synapse of innate immune cells would be an ancestor of the immunological synapse in the adaptive immune system (123).

## Author Contributions

FN, VDB, and AA contributed equally to this review.

## Conflict of Interest Statement

The authors declare that the research was conducted in the absence of any commercial or financial relationships that could be construed as a potential conflict of interest.
